# Funerary practices of cremation at the megalithic societies of South-Eastern Iberia: The cemetery of Los Milanes

**DOI:** 10.1371/journal.pone.0330771

**Published:** 2025-09-03

**Authors:** Paula Becerra Fuello, Gonzalo Aranda Jiménez, Miriam Vílchez Suárez, Sonia Robles Carrasco, Lara Milesi García, Marta Díaz-Zorita Bonilla, Christophe Snoeck, Elisavet Stamataki, Javier Lescure, Margarita Sánchez Romero

**Affiliations:** 1 Department of Prehistory and Archaeology, University of Granada, Granada, Spain; 2 Department of Historical Sciences, University of Malaga, Malaga, Spain; 3 Institute for Pre- and Protohistory and Medieval Archaeology, University of Tübingen, Tübingen, Germany; 4 Archaeology, Environmental Changes, and Geo-Chemistry Research Group, Vrije Universiteit Brussel, Brussels, Belgium; 5 Research Unit: Anthropology and Human Genetics, Department of Biology of Organisms and Ecology, Université Libre de Bruxelles, Brussels, Belgium; 6 Physical Anthropology Unit, Department of Biodiversity, Ecology and Evolution, Faculty of Biological Sciences, Universidad Complutense de Madrid, Madrid, Spain; Austrian Academy of Sciences, AUSTRIA

## Abstract

The archaeological excavations undertaken at the Chalcolithic necropolis of Los Milanes have revealed a previously unknown variability in funerary practices in the south-eastern Iberia. For the first time, a megalithic tomb housed a large funerary deposit (28,740 bone fragments) of exclusively cremated human bone remains. For a comprehensive characterization of the funerary ritual, a cutting-edge multi-proxy approach has been undertaken including the osteological study of cremated bone remains, radiocarbon chronology, Fourier-Transform Infrared spectroscopy in Attenuated Total Reflectance mode (FTIR-ATR), and carbon, oxygen and strontium isotope analyses. As a result, the cremation ritual consisted of multi-depositional events of at least 21 individuals chronologically concentrated in the first quarter of the third millennium, principally in the 28th century cal BC. The absence of charcoal/ashes in the funerary chamber and the underrepresentation of anatomical regions such as lower limb and trunk suggest that the cremation took place elsewhere and the bone remains were carefully collected and placed as secondary burial depositions. Different proxies including colour patterns, heat‐induced fractures, the presence of cyanamide in calcined bones would also suggest the cremation of principally complete corpses, burnt soon after death. The ritual of cremation coexisted with inhumations during the third millennium cal BC, suggesting a variability in the body manipulation that previously went unnoticed. Unlike inhumations, through cremation, bodies would have been reduced until being indistinguishable, transforming radically the nature of human beings and their ontological status.

## Introduction

South-eastern Iberia is one of the most traditional megalithic regions in Iberia since the end of the 19^th^ century, when Luis Siret and Pedro Flores excavated dozens of cemeteries and hundreds of tombs [1,2]. Since then, megalithic monuments have been considered the principal architectures built for ritual and mortuary purposes during the Late Neolithic and Chalcolithic periods (*ca.* 3900–2200 cal BC). Recent studies have revealed that the continuity of megalithic funerary practices was also a key feature of the Bronze Age societies (*ca.* 2200–850 cal BC) [3–8]. Megalithic monuments stand out as the most long-lasting cultural manifestation of south-eastern Iberia societies.

**Fig 1:**
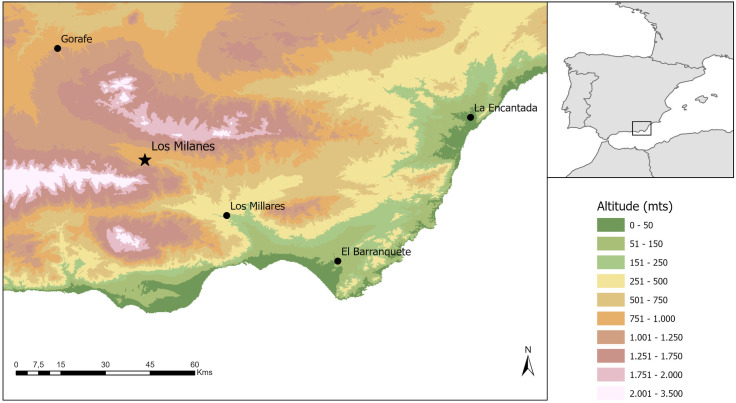
Map with the location of the main sites mentioned in the text. Printed under a CC BY 4.0 with permission from Antonio Montufo Martín.

Megalithic funerary rituals consist principally of multi-depositional events that produce complex palimpsest of stratified, fragmented and scattered bone remains. Mortuary depositions have typically been disturbed by later activities, mainly subsequent burials, making finds of articulated or semi-articulated bone remains uncommon [9]. Occasionally burned or partially burned bones have been reported associated with the unburnt bones that form the large part of the mortuary depositions. This is the case of several tombs found at megalithic cemeteries such as El Barranquete [10,11], Los Millares [2,12], La Encantada [13] and Gorafe [2,14] among others (Fig 1).

When burnt bones are present at megalithic burials in southeastern Iberia, they normally exhibit a homogeneous dark brown or black colour, indicating that they were not exposed to extremely high temperatures as the presence of calcined bones is very unusual. These remains are mostly charred, with minimal fragmentation, warping, or shrinkage. Based on these characteristics, it has been suggested that fire exposure occurred when the bones were already dry, after the bodies had fully skeletonized. Rather than being part of a structured funerary ritual, this burning has been considered as a means of cleansing, sanitizing, or purifying funerary chambers [10,12,15–19]. Recent studies propose an alternative interpretation, linking cremated bone remains to specific funerary practices associated with the reuse of older megalithic monuments during the Late Bronze Age (ca. 1300–850 cal BC) [4,5,20]. This suggests a complex and evolving funerary landscape in southeastern Iberia, where burning may have served different purposes across time and cultural contexts.

The archaeological excavations undertaken in 2023 in the megalithic necropolis of Los Milanes (Abla, Almería) have revealed a very different picture that challenges traditional explanations. For the first time, a megalithic tomb has been found containing a funerary deposit composed exclusively of cremated bone remains, a hitherto completely unknown situation in southeastern Iberia but not in other Iberian regions. Especially noticeable have been the recent findings of large deposits of cremated bones at contemporary sites such as Perdigões [21–23] and the Lácara passage dolmen [24], both located in the Guadiana basin (southwestern Iberia).

Tomb 8 at Los Milanes offers an excellent opportunity to explore whether cremation was a common chalcolithic funerary practice in south-eastern Iberia. For this purpose, a multi-proxy approach was employed, which includes the osteological study of cremated bone remains, Fourier Transform Infrared Spectroscopy (FTIR-ATR), radiocarbon ^14^C dating and carbon (*δ*^*13*^C), oxygen (*δ*^18^O) and strontium (^87^Sr/^86^Sr) isotope analyses. All these methods are used for a better understanding of three main aspects: the type of ritual process *–*primary vs. secondary deposition*–*, the chronology of funerary practices and the pre-burning conditions. As a result, a comprehensive characterisation of funerary ritual has been obtained and discussed in the context of the Iberian megalithic societies.

### Archaeological background: Los Milanes cemetery

The megalithic societies of southeastern Iberia have been studied over the past 14 years as part of a long-term research programme led by the GEA Research Group (University of Granada) (www.webgea.es). The project has focused on the study of old excavations, principally those carried out at the end of 19^th^ century by Luis Siret and Pedro Flores [6–8,25–29], as well as the development of new excavations. As a results, nine passage dolmens at the cemetery of Panoría [9,26,30–35] and three *tholos*-type tombs at Los Milanes cemetery were excavated. The Los Milanes excavation was authorised by Ministry of Culture and Sports of the Regional Government of Andalusia and was directed by Gonzalo Aranda Jiménez, one of the paper co-authors [36].

**Fig 2:**
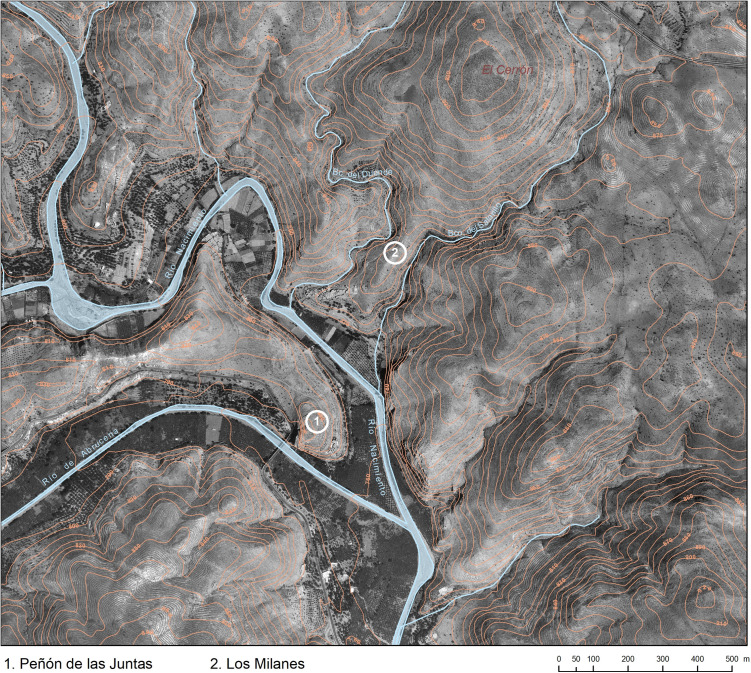
Map with the location of El Peñon de las Juntas settlement and Los Milanes cemetery. Printed under a CC BY 4.0 with permission from Antonio Montufo Martín.

**Fig 3:**
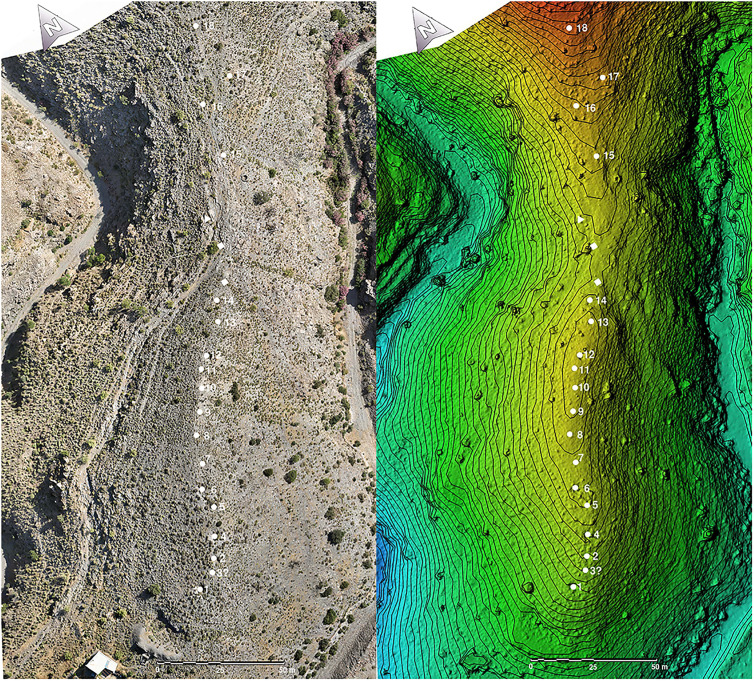
Orthophotography (left) and Digital Elevation Model (right) with the location of the 18 tombs at Los Milanes cemetery. Printed under a CC BY 4.0 with permission from José Antonio Benavides López.

The cemetery of Los Milanes together with the associated El Peñón de las Juntas settlement, both separated by the Nacimiento river (Fig 2), are in a very privileged position in the middle of a major natural corridor that links coastal with inland regions. Intensive surveys carried out in 2023 have found 18 tombs aligned almost at regular intervals in the upper areas of Los Milanes hill (Fig 3). Most of these tombs show clear evidence of looting, particularly in the form of large pits that coincide with the locations of funerary chambers. Architectural evidence supports their classification as *tholoi*, a type of tomb characterised by a circular or oval funerary chamber, with diameters usually ranging from 2 to 4 meters. They were built using dry-stone construction technique and covered by false vaults. The funerary chambers were entered through passages, normally divided into equal segments by perforated slabs. These tombs were covered by mounds formed by concentric stone walls filled with earth and small stones.

**Fig 4:**
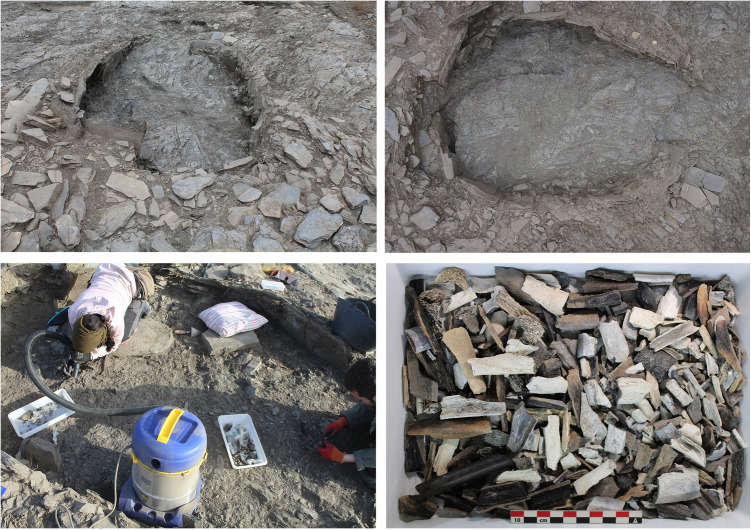
Tomb 8 at Los Milanes cemetery. Top: Tomb 8 excavated. Bottom left: excavation process; Bottom right: long bones after cleaning.

In 2023, the excavation of tombs 8, 12 and 18 was undertaken. Tomb 8 is located at the centre of the Los Milanes hill plateau, showing no evidence of looting. Only the uppermost part of two standing stones was visible on the surface. The excavation confirmed the presence of a burial chamber, oval in shape *–*3 m long and ca. 1.90 m wide*–,* partially excavated into the bedrock and formed by slate uprights (Fig 4). The corridor, though very poorly preserved, appears aligned with the longitudinal axis of the chamber and oriented toward the east. The mound consists of up to two superimposed rows of slate stones that appear to have interlocked with one another [36].

Inside the funerary chamber and passage, a single compact layer of exclusively cremated remains was found. These skeletal remains were commingled, scattered, and highly fragmented displaying clear heat‐induced alterations such as changes in colour, shrinkage, fractures and warping. Detailed archaeological excavation methods were applied, including digital technologies for recording archaeological evidence and the systematic dry sieving of all sediment using double sieves with mesh apertures of 5 and 2 mm. Associated with these remains, typical chalcolithic grave goods*–* such as idols crafted from polished animal phalanges, arrowheads, pottery fragments, burnt faunal remains, fragments of bone awls, and hundreds of small stone beads*–* have been found. The complete absence of charcoal, ashes or burning evidence within the chamber suggests that the cremation took place elsewhere and that the bone remains were carefully collected and placed inside the tomb as secondary depositions [36].

## Materials and methods

### Osteological analysis

Human remains were systematically classified according to the degree of fragmentation, with each bone fragment measured and weighed. Fire exposure of bodies leads to colour changes that are associated with different burning temperatures. Bone remains were classified into ten colour categories adapted from Ellingham et al. [37], five degrees of burning according to Carroll and Smith [38], and nine colour patterns defined by Schmidt and Symes [39] (S1 Table).

In addition to colour, heating also produces changes in bone morphology, such as warping, and fractures including longitudinal, thumbnail, step, transverse, patina and delamination, which were recorded following Herman and Bennet [40], Mayne [41], and Symes et al. [42]. Thermal alterations in teeth follow the methodologies developed by Schmidt and Symes [39] and Sandholzer [43]. The representation of anatomical regions can also be used to assess the ritual of cremation. For this purpose, burnt bone remains were first categorised into anatomical groups, then weighed, and the resulting values were compared with the expected proportions calculated via linear regression, following the method developed by Gonçalves et al. [44].

Sex estimation was recorded according to the dimorphic characteristics of the pelvis and cranium [45] as well as the measurements of talus, distal humerus, dens of the axis, mandibular condyle, patella, and radius head, following the methodology of Cavazzuti et al. [46]. Age-at-death was estimated for adults in one case based on morphological changes in the auricular surface [47] and in nine cases through dental attrition [48,49], thanks to the preservation of enamel in teeth with low degree of burning. For subadults, age-at-death was estimated according to the dental/skeletal development and epiphyseal fusion [45,50,51]. For further information, the osteological database can be consulted in the Online repository of University of Granada https://digibug.ugr.es/handle/10481/105379.

### Radiocarbon (^14^C) dating

Since the late 1990s, the radiocarbon dating of the mineral composition of cremated bones has been proven a reliable chronological method [52,53]. When bones are exposed to temperatures >600°C, all water and organic matter are destroyed and only the inorganic fraction of bone (bioapatite) remains. After burning, the bioapatite fraction still contains some carbonates that can be used for dating. That carbon represents a mixture incorporated during recrystallization from carbon of the body and principally from the fuel [54–57]. One important concern when dating the bioapatite of cremated bones consists in the potential wood-age offsets, as the carbon dated appears to be delivered mostly from the pyre fuel [58]. In those cases in which old wood, coal, or even peat are used in the pyre, the radiocarbon measurement will be affected by “old-wood effect” [54,57,59,60]. The degree to which radiocarbon measurements are affected will depend on the age of the wood-fuel used [60]. In south-eastern Iberian, palynological analyses have revealed an arid environment and a landscape dominated by steppe vegetation during the Chalcolithic period [61–63]. At that time, arboreal cover was drastically reduced, while the Mediterranean shrub increased quickly. Although the use of significantly older wood cannot be ruled out, it seems unlikely, as the most available fuel would have been branch wood, brush, and small trunks. If that was the case, a very limited wood-age offset is expected.

Careful selection of fully calcined bones is essential for obtaining reliable radiocarbon measurement [56,58]. Calcined bones with a uniformly white colour were selected for dating. Between the different anatomical regions identified in the osteological analysis, the left humerus sums the largest number of calcined bones ensuring that no individual is dated twice. Seven samples (six left humeri from adults and one femur from a subadult) were selected. Five of them also underwent FTIR analysis (see below), and their IRSF, C/C and CN/P values confirms that they are fully calcined bones [64]. Samples were measured at the Centre for the Isotope Sciences (SUERC) using Accelerator Mass Spectrometry (AMS) (following Dunbar et al. [65]). Radiocarbon dates were calibrated using OxCal v4.4.4 software [66–68] with the IntCal20 atmospheric curve [69]. Calibrated ranges endpoints are rounded to the nearest 10 years when the error was ≥ 25 years, and to the nearest 5 years when the error was **<* *25 years [70,71]. Bayesian models and Kernel Density Estimation plots were generated using OxCal program v4.4.4 [66,67].

### Fourier-Transform Infrared spectroscopy in Attenuated Total Reflectance (ATR) mode analysis (FTIR-ATR)

The study of heat-induced structural and chemical changes on cremated bone has improved since the development of methodologies such as the Fourier-Transform Infrared Spectroscopy (FTIR) in Attenuated Total Reflectance (ATR) mode [72–77]. Different FTIR indices have been measured to estimate the burning conditions during cremation such as temperature and combustion in reducing environments (S2 Table) [76,78–82]. 21 samples were selected according to the minimum number of individuals (MNI) based on the count of left petrous bones. As most of these samples were not fully calcined according to their coloration, five more samples of calcined long bones were also analysed to complete the sampling strategy.

The 26 selected bone fragments (ca. 200 mg) for FTIR-ATR and carbon and oxygen isotopes analyses were mechanically and chemically pre-treated at the Archaeology, Environmental Changes, and Geochemistry (AMGC) research Group at Vrije Universiteit Brussel (VUB) in Belgium, to remove secondary carbonates and any post-burial contamination. Firstly, all the samples were mechanically cleaned through drilling, following the method outlined by Stamataki et al. [76]. Subsequently, a chemical cleaning with acetic acid was performed following the protocol established by Snoeck et al. [83] and McMillan et al. [84].

For FTIR-ATR measurements, 6 mg of sieved bone powder (25−50μm fraction) was used following the protocol of Stamataki et al. [76]. All the samples were analysed in triplicates (ca. 2 mg for each measurement) and the infrared indices reported in this study represent the average of three measurements and standard deviation, both included the S2 Table. FTIR analysis was performed at VUB at the AMGC research unit using a Bruker Vertex 70v FTIR spectrometer under vacuum (spectral range: 4000–400 cm-1; number of scans: 32; spectral resolution: 4 cm-1; mode: absorbance). Infrared spectra were analysed using OPUS 7.5 software and all the indices were calculated after the baseline correction as described in Stamataki et al. [76].

### Carbon and oxygen isotope analyses

Cremation conditions such as the combustion environment, oxygen supply and fuel can also be approached using carbon and oxygen isotope analyses. Carbon isotope values (δ^13^C) have been principally associated with the wood fuel of the pyre, but also with the interchange of carbon with endogenous collagen, garments and other pyre-goods [55,60,80,85]. In this study, emphasis was placed on the carbonate fraction of bioapatite, as its δ^18^O value is measured simultaneously with δ^13^C during stable isotope analysis, offering a practical and integrated proxy for assessing burning conditions. When interpreted alongside carbon isotopes and infrared indices, oxygen isotope data provide valuable insights into burning intensity (e.g., [56,76,82,86]) oxygen availability during combustion (e.g., [80]), seasonality and weather conditions [87], and body and pyre management practices [80]. Together, stable carbon and oxygen isotope analyses offer a powerful and complementary approach for reconstructing the conditions under which cremation was performed in past societies. The sampling strategy focused on the same 26 samples previously analysed.

For the analysis of carbon and oxygen isotopes, 30 mg of bone powder (ca. 15 mg for each of the duplicates) was used following the protocol as described by Stamataki et al. [76]. The released CO_2_ was analysed with a Nu Perspective IRMS (Isotope Ratio Mass Spectrometer) from Nu Instruments (Wrexham, UK) coupled with a Nu GasPrep automatic gas bench at the VUB. International standards IA-R022 (n = 4) (δ^13^C = −28.6‰ and δ^18^O = −22.7‰), IAEA-603 (δ^13^C = 2.5‰ and δ^18^O = −2.3‰) (n = 3) were used to calibrate the isotopic data, and the analytical precision was better than ± 0.09‰ (δ^13^C) and ± 0.09‰ (δ^18^O) (1SD) based on repeated measurements of in-house cremated bone standard CBA (n = 4; see De Winter et al. [88]).

### Strontium isotope analyses

Strontium isotope analysis can also be used as indirect proxy of the ritual practice of cremation [89] but can only be used on calcined bones. Indeed, calcined bones are resistant to diagenetic contamination from soil [55], which means that strontium signatures of bones provide reliable information of mobility patterns [81,83,90]. Due to the low number of fully calcined samples representing distinct individuals, strontium isotope and concentration analyses could only be performed on five calcined long bones, which were also selected for radiocarbon dating. All isotopic analysis were carried out at the Vrije Universiteit Brussel (VUB) following Snoeck et al. [83] and Gerritzen et al. [91] for the isotope analyses and Boonants et al. [92] for the concentrations.

Pre-treated calcined bone powder (c. 15 mg) was placed in a Teflon beaker and dissolved with 1mL 14M subboiled HNO_3_. Once fully dissolved, the samples were left to dry overnight on a hotplate at ca. 100°C. Strontium was extracted from the samples and purified through column chemistry using ion exchange resin (Sr-Spec, Triskem), following the protocol described in Snoeck et al. [83]. Subsequently the samples are evaporated to dryness and redissolved in a 1.5 mL 0.05 M HNO_3_ running solution. The ^87^Sr/^86^Sr measurement as well as the determination of strontium concentrations ([Sr]) were performed on a Nu Plasma 3 MC-ICP-MS (PD017 from Nu Instruments, Wrexham, UK) at AMGC (VUB). During this study, repeated measurements of the NIST SRM987 standard yielded ^87^Sr/^86^Sr = 0.710271 ± 0.000033 (2SD; n = 5), which is consistent with the mean value of 0.710252 ± 13 (2SD for 88 analyses) obtained by TIMS (Thermal Ionization Mass Spectrometry) instrumentation [93]. All the sample measurements were normalised using a sample-standard bracketing method with the recommended value of ^87^Sr/^86^Sr = 0.710248 [93]. Procedural blanks were considered negligible (total Sr (V) of max 0.02 versus 10V for analyses, i.e., ≈ 0.2%). For each sample the ^87^Sr/^86^Sr value is reported with a 2SE error (absolute error value of the individual sample analysis – internal error). To obtain [Sr], the voltage obtained for ^88^Sr for each sample was compared to the voltage obtained for the 300ppb solution of NIST SRM 987. During the course of these analyses the matrix matched standard NIST SRM1400 (bone ash) was used and returned mean ^87^Sr/^86^Sr values of 0.713124 ± 0.000002 (2SD; n = 4) and [Sr] of 222 ppm ± 7%RSD (n = 4). This value is close to the long term ^87^Sr/^86^Sr values of 0.713957 ± 0.000035 (2SD; n = 233) obtained by Gerritzen et al. [91] and the reported [Sr] of 249 ± 7 ppm.

### Statistical analysis

Statistical analyses were carried out in R 4.4.2 [94]. Figures were also generated in R, using the packages ggplot2 and viridis [95,96]. The statistical tests used were χ2, two-sided Wilcoxon test, Kruskal-Wallis and Permutational Multivariate Analysis of Variance (PERMANOVA) [97].

## Results

### Osteological analysis of cremated bones

Tomb 8 contained 28,740 bone fragments (12.63 kg) and 1,209 dental remains, deposited at the University of Granada facilities for their study. The skeletal collection is characterised by a high degree of fragmentation, with 73.8% of the bone fragments measuring less than 20 mm in size, and only 15 bones exceeding 90 mm. Due to this fragmentation, 38.4% of bones remains were unidentified and 31.7% could only be classified as fragments of the diaphysis of long bones. Nevertheless, all anatomical elements have been recorded, standing out the presence of small bones such as sesamoids, and distal foot and hand phalanges. The MNI was 21, based on the left petrous bone. A MNI of three non-adults has also been identified based on those teeth 44 whose roots were still at formation stages. These non-adults are not summed to the MNI, as the age of the left petrous bones could not be determined. Three individuals were possibly female according to the measurement of dens axis and the supra-orbital margin features, and three possible males based on the talus measurements [45,46].

**Fig 5:**
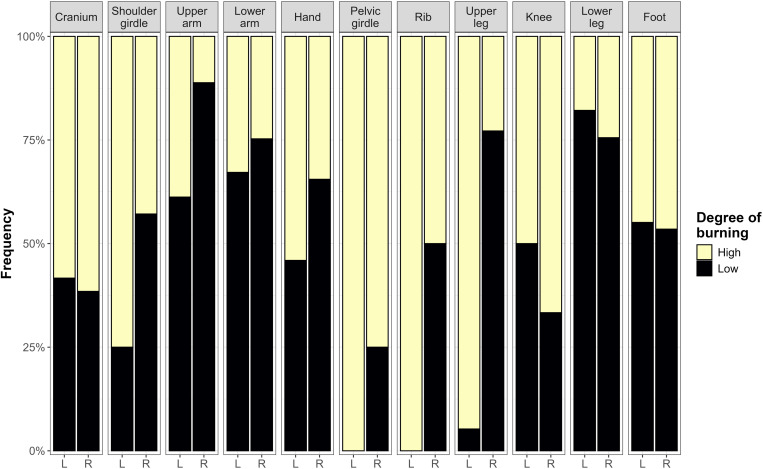
Variability of burning degree by anatomical regions. Black colour clusters degrees 1, 2 and 3 and yellow 4 and 5.

The bone remains displayed heterogeneous colours from brown, black, grey, blue-grey to white. Based on this variability, five degrees of burning have been established, ranging from slightly burned to >50% of the bone fragment being calcined (see categories details on S1 Table). As a result, 27.1% of the skeletal remains were concentrated in degree 1, 6.5% in degree 2, 6.5% in degree 3, 12.2% in degree 4, and 47.7% in degree 5. For the analysis of burning patterns by anatomical regions, these degrees have been clustered in two categories: high degree of burning includes categories 4 and 5, and low degree of burning categories 1, 2 and 3 (Fig 5). The difference between the two burning categories is that, under high-degree burning all bones are at least partially calcined, indicating exposure to temperatures above 650ºC. These two burning categories have been studied for 11 anatomical regions (n = 1136) (Fig 5). As a result, the left side of the bodies (n = 406) reached higher degrees of burning than the right side (n = 730), a difference which is statistically significant (Pearson’s chi-squared test = 38.872, with a p-value= < 0.0001). The left side reached a higher degree of burning in left anatomical regions such as shoulder girdle, left upper and lower arms, hands, ribs and upper leg.

**Fig 6:**
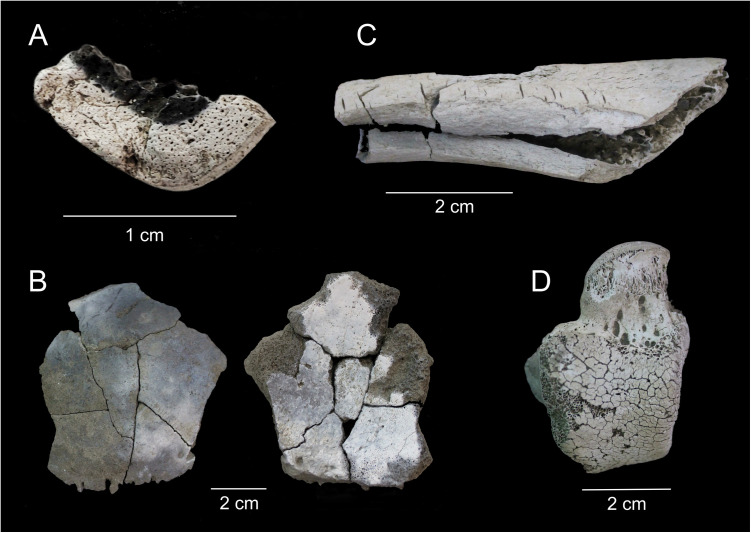
Heat-induced changes found at Los Milanes. A: colour pattern with higher degree of burning in the external cortical of a long bone. B: colour pattern with higher degree of burning in the internal versus the external surface of a skull fragment. C: warping on a left humerus. D: left talus with patina*.*

Colour variability also revealed different patterns that were observed in 18.2% of the total bone assemblage. Eight colour-pattern categories have been identified (see S1 Table), principally concentrated in long bones and crania (Fig 6 and Table 1). In the former case, a higher degree of burning was found in the external cortical surfaces of 98.3% of the fragments, which clearly indicate that long bones were exposed to fire before fragmentation. This pattern is less clear in cranial bones, as evidence of higher temperatures on the external versus internal surfaces and vice versa appear in the same proportion (47.8% *vs*. 52.2% among cranial fragments exhibiting patterned coloration).

**Table 1:** Colour patterns found at Tomb 8 (n = 5238).

Categories	Colour pattern	N	%
1	“Sandwich” effect	935	17,85
2	Long bones: Higher temperature outside and lower inside	3657	69,82
3	Long bones: Lower temperature outside and higher inside	60	1,15
4	Cranium: Higher temperature outside and lower inside	258	4,93
5	Cranium: Lower temperature outside and higher inside	281	5,36
6	Cranium: Suture with a different coloration	11	0,21
7	Heat line	29	0,55
8	Others	7	0,13

Of the 28,740 bone fragments, 28.4% shows clear heat‐induced fractures, principally delamination (37.4%), step fracture (27%), patina (19.1%), longitudinal (9.6%), transverse (6.1%) and thumbnail fracture (0.5%). Warping also appears in the 3.2% of bones, mostly concentrated in long bones.

**Fig 7:**
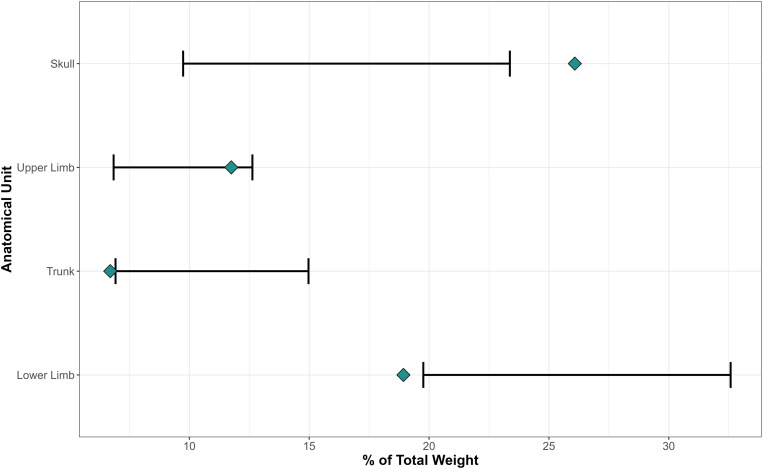
Proportional mass of anatomical categories (blue rhombuses) compared with the expected proportions (black lines) calculated via the linear regression developed by Gonçalves et al. [44].

Proportional mass of five anatomical categories (cranium, lower limb, upper limb, trunk and unidentified) was used to assess skeletal representation. Bones are weighted according to these categories and compared with the expected proportions calculated via the linear regression developed by Gonçalves et al. [44]. The bone assemblage of Tomb 8 sums 12,63 kg distributed as follows: cranium 3.29 kg (26.1%), lower limb 2.39 kg (18.9%), upper limb 1.48 kg (11.7%), trunk 0.84 kg (6.7%) and unidentified 1.32 kg (10.4%). Fig 7 shows these proportions in relation to the expected intervals. The upper limb falls within the estimated values, while the lower limb and trunk are underrepresented and the cranium overrepresented.

### Radiocarbon dating

The seven samples (six left humeri from adults and one femur from a subadult) selected for radiocarbon dating were successfully measured at the Centre for the Isotope Sciences (SUERC) using Accelerator Mass Spectrometry (AMS) (Table 2). The radiocarbon series are very consistent, concentrating all dates in comparable chronological intervals. None of them appears as an archaeological outlier, and all dates match perfectly with the typological chronology of the associated grave-goods [27].

**Table 2:** Radiocarbon dates, strontium isotope values, strontium concentrations, and carbon isotope signatures from the sampled individuals at Tomb 8.

Lab. Code	BB-LAB Code	Type of material	Radiocarbon Age BP	Calibrated date (68% confidence) cal BC	Calibrated date (95% confidence) cal BC	δ^13^C (‰ – VPDB) AMS	δ^13^C (‰ – VPDB) IRMS	^87^Sr/^86^Sr	2 SE	[Sr] (ppm)
SUERC-128117	PBF022	Left Humerus	4227 ± 22	2895−2780	2900−2705	−22.9	−24.0	0.712486	0.000012	209
SUERC-128114	PBF023	Left Humerus	4165 ± 22	2875−2695	2880−2635	−16.9	−19.3	0.712752	0.000008	186
SUERC-128116	PBF024	Left Humerus	4162 ± 22	2870−2675	2880−2635	−19.3	−18.9	0.712301	0.000011	227
SUERC-128113	PBF025	Left Humerus	4134 ± 22	2860−2630	2870−2585	−19.6	−19.9	0.711269	0.000012	214
SUERC-128119	PBF026	Femur	4185 ± 22	2880−2700	2885−2675	−19.6	−22.8	0.711677	0.000010	189
SUERC-128115		Left Humerus	4062 ± 22	2625−2500	2840−2490	−14.4				
SUERC-128118		Left Humerus	4104 ± 22	2845−2815	2860−2575	−14.7				

**Fig 8:**
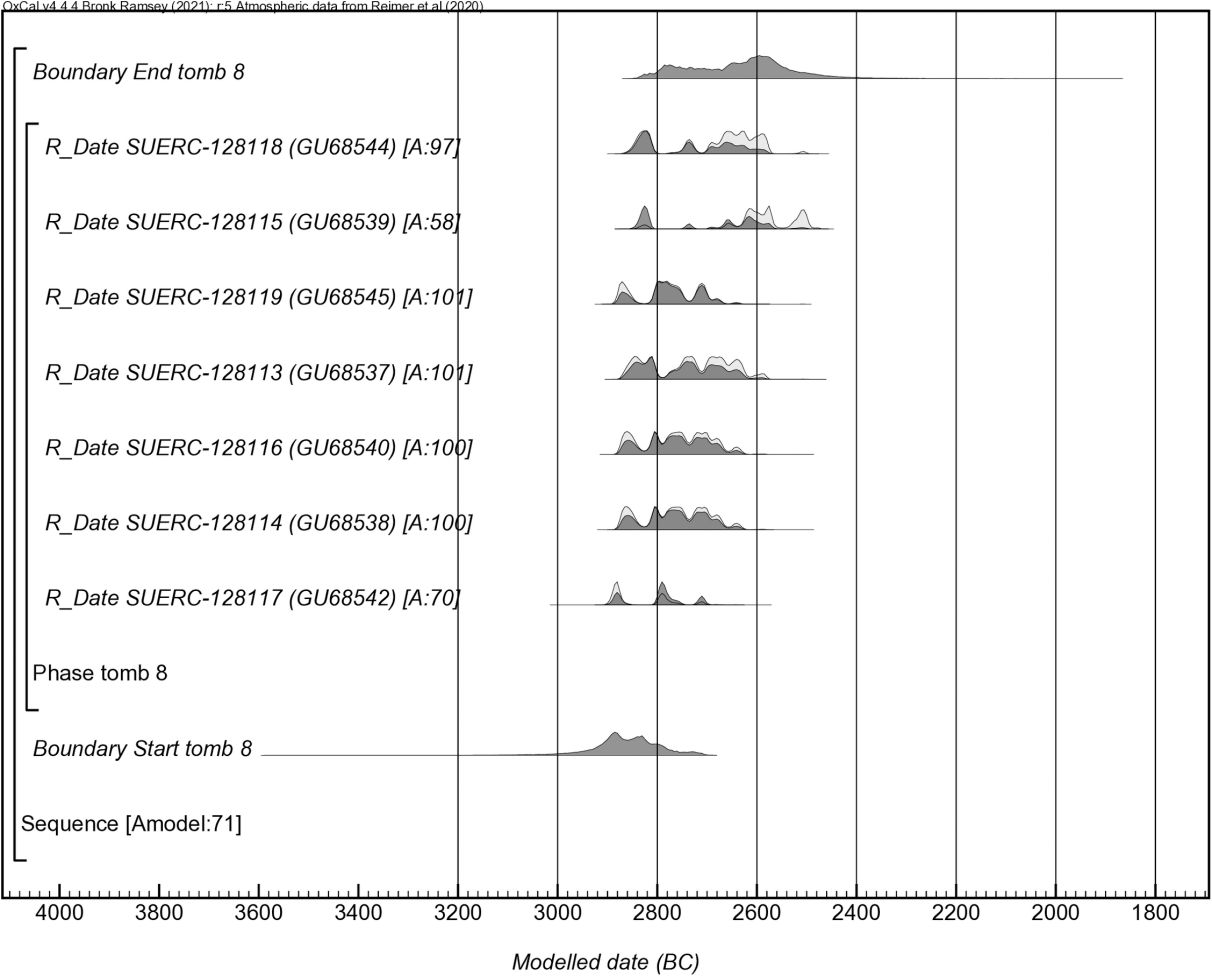
Bayesian model of radiocarbon dates from the Tomb 8 at Los Milanes cemetery.

**Fig 9:**
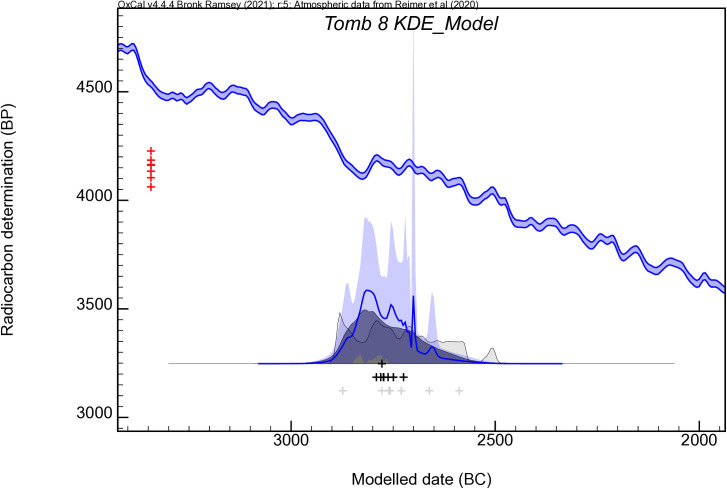
KDE-modelled distribution of dates from the Tomb 8 at Los Milanes cemetery.

A Bayesian model was built, clustering all dates in a simple bounded phase. According to this model (index of agreement of Amodel = 71) (Fig 8) the first bodies would have been deposited between *2990 and 2710* cal BC (*95% of probability; boundary Start*) or between *2910 and 2800* cal BC (*68% of probability*), and the last between 283*0 and 2460* cal BC (*95% of probability, Boundary End*), or possibly in *2790–2540* cal BC (*68% of probability*). The difference between these two distributions suggests a period of mortuary activity between 45 and 220 calendar years (*68% probability Span)*. Assuming a period of 25 years for a generation (following Whittle et al. [98]), just a few generations – between two and eight generations– were buried in Tomb 8. The short period of use is also supported by the statistical test of consistency as five of the seven dates pass the test (T = 8.2; df = 4; T’ (5%) = 9.5) [99], which means temporally close cremation events. If all radiocarbon dates are included in a KDE model (Kernel Density Estimation) (Fig 9), mortuary activity appears principally concentrated in the 28th century cal BC.

### FTIR-ATR analysis

**Fig 10:**
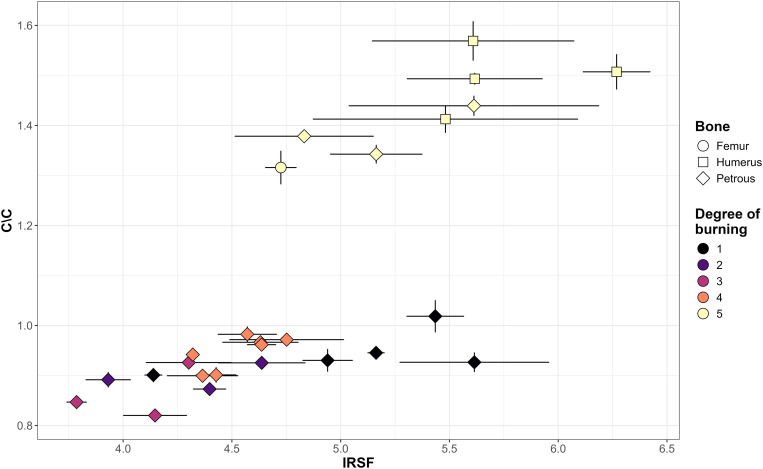
Scatterplot of IRSF to C/C values according to the degree of burning and the type of bone. The error bars indicate the 1st and the 3rd quartiles.

Infrared Splitting Factor (IRSF) values range from 3.79 to 6.27, with a mean of 4.82 and a standard deviation of 0.62 and carbonyl-to-carbonate (C/C) content ranges from 0.82 to 1.57 with a mean of 1.08, and standard deviation of 0.25 (S2 Table). Both indexes would indicate that the degree of crystallinity and the carbon content in bone is highly variable, which is consistent with the different degrees of burning identified. Fig 10 shows the relationship between IRSF and C/C values according to the degree of burning and the type of bone. Two main aspects can be highlighted. The C/C value of calcined bones (Degree 5) ranges from 1.57 to 1.32 clearly separate from the other 4 degrees of burning that concentrate between 0.82 and 1.02 (the difference between these groups were evaluated through a Permutational Multivariate Analysis of Variance (PERMANOVA) (R2 = 0.69 p-value: < 0.0001). Wilcoxon tests were carried out independently for IRSF and C\C, with p-values of less than 0.001 in both cases (0.0006 and 0.000001, respectively). The opposite trend appears when IRSF values are considered as calcined bones (Degree 5) ranging from 4.73 and 6.27 and the other 4 degrees between 3.79 and 5.61. Both intervals overlap between samples with different degrees of burning. High crystallinity values are expected in calcined bones (Degree 5) but not in slightly burned bones (Degree 1). In these cases, diagenetic processes could explain the increase of crystal size in bioapatite [100]. In the bone assemblage of Tomb 8, weathering is the taphonomic factor that would explain these crystallinity values as it appears significantly associated with slightly burned bones (n = 2792) (Pearson’s chi-squared, Yates continuity correction with a p-value of <0.0001). For a better understanding of structural and chemical changes in cremated bones, IRSF values should be analyzed together with C/C content and the macroscopical evaluation of the degree of burning.

**Fig 11:**
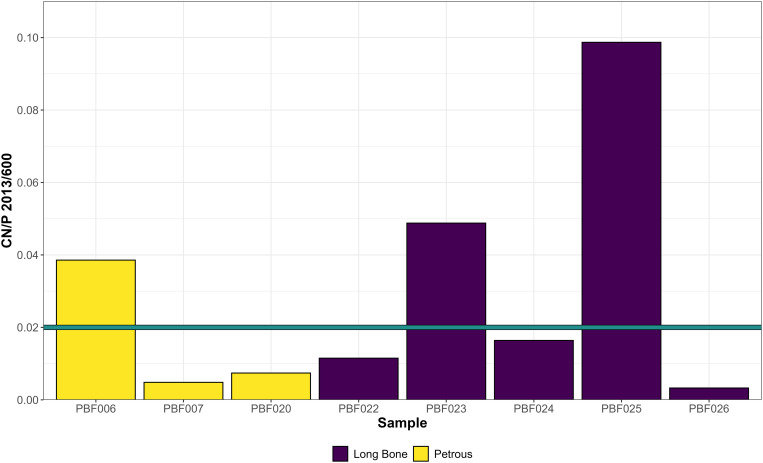
CN/P ratios of the eight calcined samples. Blue line indicates the > 0.02 threshold (Salesse et al. 2021 [80]).

Infrared Spectroscopy (FTIR-ATR) can also determine the cyanamide to phosphate ratio (CN/P) on calcined samples. The cyanamide is suggested to be formed under more reducing conditions [74]. Of the eight samples with a degree of burning 5, three show ratios >0.02 (Fig 11) [76,80]. Interestingly, these three bones also display evidence of warping, which means that the temperature was high enough to produce changes in bone morphology. The appearance of cyanamide has been linked to low oxygen availability during burning [64] which could be potentially tied to the presence of shrouds wrapped around the individuals during cremation and also to body position on the pyre [80].

### Carbon and oxygen isotope analyses

**Fig 12:**
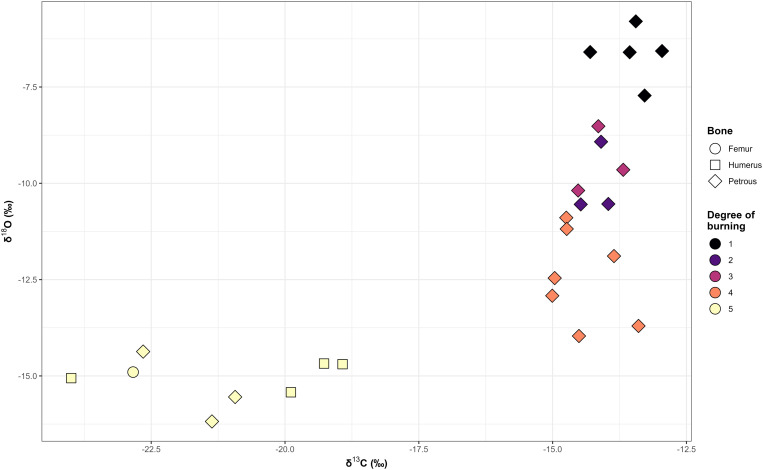
Scatterplot of δ^13^C to δ^18^O values according to the degree of burning and the type of bone.

Carbon and oxygen isotope ratios range from −24 ‰ to −13 ‰ and from −16.2 ‰ to −5.8 ‰ respectively (S2 Table). When they are combined with the five degrees of burning, two different patterns appear (Fig 12). Firstly, δ^18^O values decrease gradually as the temperature increases, an association previously noted by Snoeck et al. [56]. Secondly, δ^13^C values cluster in two groups, the first four degrees of burning range from −15 ‰ to −13 ‰ and Degree 5 from −24‰ to −18.9‰, a difference statistically significant (Wilcoxon test, *p-value* = < 0.0001). Carbon and oxygen values from calcined samples (δ^13^C: −24‰ to −18.9‰, δ^18^O: 16.2‰ to −14.4‰), do not show significant differences among them (Kruskal-Wallis test, *p-value* = 0.3998 and *p-value* = 0.7575 respectively), suggesting a similar type and amount of fuel and oxygen availability.

### Strontium isotope and concentration analyses

**Fig 13:**
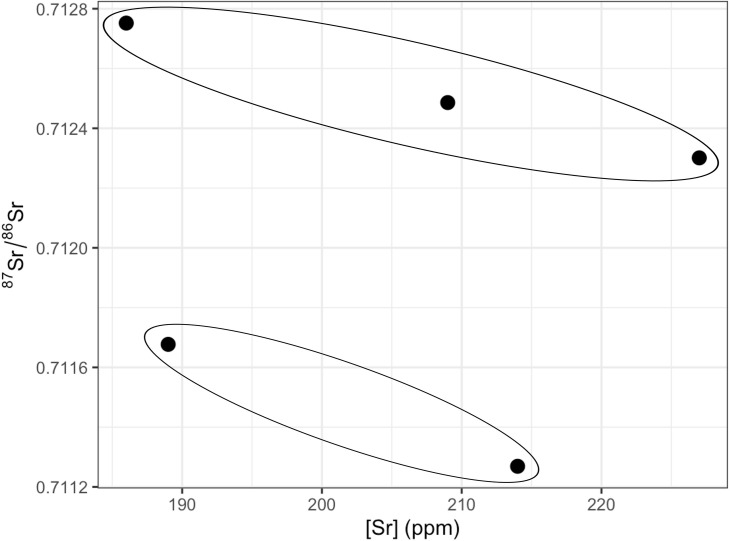
Scatterplot of ^87^Sr/^86^Sr ratio to [Sr] values of five calcined humeri. The two circles represent the two identified groups.

**Fig 14:**
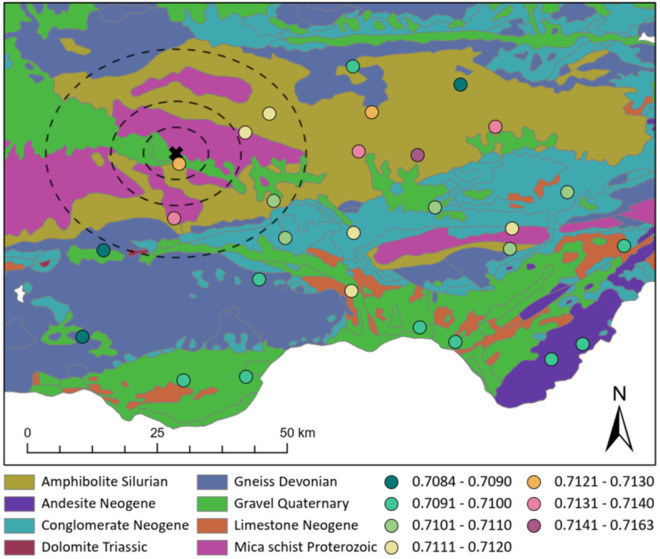
Geological map based on Mapa Geológico de la Península Ibérica, Baleares y Canarias a escala 1:1.000.000, 2015 [103] (https://info.igme.es/cartografiadigital/geologica/Geologicos1MMapa.aspx?Id=Geologico1000_(2015)&language=en), using the lithology and lower geological age categories. The cross represents the location of Los Milanes and the coloured dots represent the locations of plants collected and analysed by Frank et al. [102] with the colour showing their strontium isotope ratios. The circles are 5, 10 and 20 km around the site. Printed under a CC BY 4.0 with permission from Hannah F. James.

The strontium isotope ratios of the five calcined bone fragments range from 0.7112 to 0.7128 and the concentrations ([Sr]) range from 186 to 229 ppm (Fig 13). While the number of samples is limited due to the lack of suitable fragments for analyses, these results provide insights into the possible origin of those buried at the site. The difference between the lowest and highest ^87^Sr/^86^Sr value is 0.0016, which confirms that these bone fragments belong to different individuals [89,101] and that they likely consumed foods originating from different areas. The strontium isotope ratios can be clustered in two groups according to the ^87^Sr/^86^Sr (Fig 13) with two samples having lower values (0.7112 and 0.7117) and the other three with higher values between 0.7123 and 0.7128, potentially suggesting they might belong to two different groups. Still, these ⁸⁷Sr/⁸⁶Sr values fall within the local range (average (± 2SD) bioavailable ⁸⁷Sr/⁸⁶Sr within 20 km of the site based on 5 plant samples: 0.7118 ± 0.0021 [102]; Fig 14), suggesting the individuals are all local but sourcing foods from different zones around the site.

## Discussion

Cremated remains in Late Neolithic and Chalcolithic megalithic and collective burials are also present in different Iberian regions [22–24,104–108]. As burned skeletal remains typically appear as a small part of the bone assemblages, discussions have been principally focused on their accidental or intentional nature. Most accounts have emphasised that bone remains were burned once fully skeletonised [17,105]. Los Milanes together with recent findings at the Perdigões site [23] and at Lácara dolmen [24] show a very different picture involving large deposits of burnt skeletal remains that display complex heat‐induced modifications.

Tomb 8 at Los Milanes comprises a large deposit (12.6 kg) of exclusively burned human remains of at least 21 individuals. The absence of charcoal, ashes or burning evidence in the funerary chamber suggests a secondary deposition, which also agrees with the skeletal representation of different anatomical categories. The underrepresentation of the lower limb and trunk is consistent with the expected bone loss during gathering from the pyre before the final deposition in the tomb. Nevertheless, the overrepresentation of the cranium together with the outstanding presence of labile joint bones, mainly sesamoid and complete phalanges of feet and hands, also suggest a carefully gathering. Both elements, secondary depositions and thorough bone recovering have also been highlighted as key funerary practices at Lácara dolmen [24] and at the different cremated deposits *–*pits, open air and cist grave*–* found at Perdigões [21–23].

The radiocarbon series obtained at Tomb 8 suggests that individuals would have not died at the same time, which means that the deposition of cremated bones could have occurred at different funerary events. Nevertheless, chronological models would also indicate a short period of use, spanning only a few generations concentrated principally in the 28th century cal BC. The only Iberian radiocarbon series comparable, also based on cremated human bone remains, has been obtained from Perdigões [21,109]. Five dates located cremation rituals in the third quarter of the third millennium cal BC cal BC, which contrasts with Tomb 8 dated mainly in the first quarter of the third millennium. The radiocarbon series at Perdigões and Los Milanes would confirm that the ritual of cremation was an important and widely spread funerary practice during the third millennium cal BC in southern Iberia.

It seems clear that human remains were intentionally burnt in unknown places and cremated in different events before the final deposition in Tomb 8. At this point, a key aspect consists in elucidating the pre-burning conditions of the remains. Were bones cremated when they were dry and skeletonized or still with flesh, shortly after death as a complete corpse? Combining several proxies that include colour changes, fractures such as thumbnails, warping, proportional mass of different skeletal regions and bone frequency, recent accounts agree that cremations occurred principally in fleshed human remains, although not exclusively, as the burning of skeletonised remains cannot been ruled out [21–24,108]. The study of bone remains from Los Milanes supports this interpretation, emphasizing that cremation occurred mostly on complete corpses shortly after death.

Human bones from Tomb 8 appear as a highly fragmented collection that displays heterogeneous coloration, indicating that bodies were exposed to different temperatures. This variability would have affected anatomical regions differentially, as the left side of the bodies reached higher degrees of burning than the right side. This pattern suggests a deliberate left side position of the bodies on the funerary pyre, which is very consistent with the ritual pattern found in others European sites [110] and more interestingly at the near megalithic cemetery of Panoría [111,112]. Of the 21 individuals found at Panoría in articulated and flexed position, 17 (81%) were lying on the left side. At Los Milanes, this pattern is also consistent with the cremation of complete corpses, because it is very unlikely that dry and disarticulated bones would exhibit such significant side-specific differences. As it has also been found in case-studies such as Perdigões [23], at Los Milanes higher degrees of burning were found in the external cortical of long bones that would imply that bones were exposed to fire before fragmentation, likely as whole bodies. Nevertheless, this pattern is less clear in the cranial fragments as evidence of burning at higher temperatures also appear in the internal surfaces, potentially indicating different degrees of body decomposition.

Heat-induced fractures and warping are also key features of cremated bone remains that have traditionally been associated with the cremation of complete corpses. However, recent studies point out that this relationship is inconclusive, particularly in the typical thumbnail fractures that have also been found in the burning of dry bones [113,114]. At Tomb 8, although thumbnail fractures are found, the most common fractures are patina and delamination, which are related to the loss of water during heating. As DeHaan and Nurbakhsh [115] have claimed, these kinds of heating-induce changes occur on green bones as temperature increases spalling of their moist surface, a process not observed in dry bones.

The structural and chemical alterations (FTIR-ATR) of cremated bone remains from Tomb 8 show a variable degree of crystallinity, organic content and the presence of cyanamide. Isotope values would confirm a variable combustion environment that would have produced a large variability in the exposure of bodies to fire. The large amount of non-fully calcined bones in the assemblage (52.3%) highlights a burning condition that could be linked to small pyre, insufficient access to suitable wood fuel, short duration of combustion, low oxygen availability, or changing environmental conditions. Carbon and oxygen isotopic data, together with bone coloration, both indicate highly variable firing conditions and intensities, which is consistent with other European case studies [76,82]. As previous studies [64,116] have noted, bone coloration is a subjective observation influenced by both temperature and exposure time, so the combination of the macroscopic evaluation with carbon and oxygen analyses, strengthens the results of the observed degrees of burning. Also, the values of calcined samples recently published for the Italian site of San Valentino (δ^13^C values from −24.7 to −15.1 and δ^18^O values from −20.9 to −13.8 [82] and of four Belgian sites (Velzeke, Blicquy, Grand Bois and Herstal) whose δ^13^C values range between − 28.4 and −17.1‰ and δ^18^O values from −26.2 to −14.2‰ [76], show comparable variability with our calcined samples. This could reflect that the limited control of the combustion environment during cremation was a common pattern between different European prehistoric societies.

The presence of cyanamide-to-phosphate ratio (CN/P) in those fully calcined bones can be associated with lower oxygen availability during burning, which is in line with variable cremation settings. The cyanamide could potentially also be linked to the presence of garments worn by the corpses during cremation [80].

As unburned bone can be prone to diagenesis [117–120], if the bones were burned dry after having been in contact with soil for a certain amount of time, one could expect the strontium isotope ratios to show limited variability and higher [Sr] concentrations due to diagenetic exchange. However, [Sr] are similar to those seen in other Iberian sites [121,122]. Furthermore, the difference in the strontium isotope ratios of the five calcined diaphyses ranging from 0.7113 to 0.7128 suggests very limited diagenetic exchange. Instead, while the obtained ratios fall within the local baseline, the difference in values suggest they did not all source their food from the exact same location. Strontium isotope values are consistent with a very limited diagenetic exchange and the use of different intake sources, which indirectly suggest that the bodies were not cremated post-skeletonization in contact with the soil. All different proxies previously discussed point out a ritual of cremation characterized mostly by complete corpses, exposed to fire shortly after death.

## Conclusions

For the first time in Iberia, a complete cremated bone collection has been fully excavated and analysed, including the study of each of the 28,740 bone fragments and 1,209 dental remains. Besides the traditional osteological characterisation, the ritual of cremation has also been approached through carbon, oxygen and strontium isotope analysis, Fourier-Transform Infrared spectroscopy and radiocarbon dating. The combined analysis of these multi-proxies improves our understanding of the ritual practice of cremation, especially of the more elusive pre-burning conditions.

Tomb 8 at Los Milanes has revealed a previously unknown variability in funerary practices in south-eastern Iberia. For the first time, a large deposit of cremated human bones can be firmly dated to the Chalcolithic period, in the first quarter of the third millennium, principally in the 28^th^ century cal BC. Tomb 8 housed multi-depositional ritual events of burnt skeletal remains of at least 21 individuals associated with typical Chalcolithic grave-goods including arrow heads, pottery vessels and phalanx “idols”. Human remains were exposed to fire in pyres outside the funerary chamber, mostly as complete corpses. As a result, two main aspects can be settled. Firstly, the cremation was an intentional chalcolithic funerary ritual in south-eastern Iberia, as it has been found in other contemporary Iberian sites. Secondly, the cremated remains found at different south-eastern necropolises should be re-evaluated in the light of these new findings. If the burnt remains were chalcolithic deposits or/and the result of later funerary practices of reuse needs to be clarified.

The ritual of cremation appears for the first time in south-eastern Iberian as a mortuary practice during the third millennium cal BC, introducing a previously unnoticed variability in body manipulation. Inhumation rituals as primary or secondary depositions coexisted with cremations. Both rituals share the necropolis as a ceremonial space, the *tholos* as a funerary monument and similar material expressions as grave-goods. Ritual diversity seems to be principally emphasised through how human bodies are treated and manipulated. Through cremation human bodies were reduced to very small, sometimes indistinguishable, bone fragments. Even bone coloration changes dramatically after burning. On the contrary, through inhumation human bodies sometimes preserve their anatomical connections [9–11,34,111,112], and even when bones are commingled, their anatomical morphology is normally distinguishable. It is also usual to find special care to specific anatomical parts such as skulls that sometimes appear concentrated in particular areas of funerary chambers [9,112,123,124]. Inhumation and cremation rituals produce very different effects on human bodies. Then, why south-eastern megalithic communities choose one ritual over the other? At the current state of the art, it is very speculative to approach this discussion as more case studies are needed for comparative purposes. It should be noted that the cremated remains discussed in this paper are a novelty for the Chalcolithic societies in south-eastern Iberia. Although it is obvious that further research is needed for a better understanding of cremations and their relationship to inhumations, the variability of body transformation and manipulation emerges as a key aspect of funerary rituals of the Iberian Chalcolithic societies.

## Supporting information

S1 Table Categories of colour, colour pattern and fracture. Ten colour categories adapted from Ellingham et al. [36], nine colour patterns defined by Ellingham [37], Schmidt and Symes [39], Herman and Bennet [40], Mayne [41].
(DOCX)

S2 Table Results of FTIR-ATR, δ13C, δ18O, with all the FTIR-ATR indices calculated, degrees of burning, colour and presence of warping. (XLSX)
