# Supplementary material for: Funerary practices of cremation at the megalithic societies of South-Eastern Iberia: The cemetery of Los Milanes
Source: PLoS One. 2025 Sep 3;20(9):e0330771. doi: 10.1371/journal.pone.0330771 (PMC12407489; doi:10.1371/journal.pone.0330771)
Supplement: S1 Table — Ten colour categories adapted from Ellingham et al. [36], nine colour patterns defined by Ellingham [37], Schmidt and Symes [39], Herman and Bennet [40], Mayne [41]. (DOCX) [file pone.0330771.s001.docx]

**S1 Text. Categories of colour, colour pattern, degree of burning and fracture.** Ten colour categories adapted from Ellingham et al. [36], degree of burning according to Carroll and Smith [37], nine colour patterns defined by Schmidt and Symes [38] and fractures following Herman and Bennet [39], Mayne [40], and Symes et al. [41].

| Categories | Colour | Colour pattern | Degree of burning | Fracture |
| --- | --- | --- | --- | --- |
| 1 | Yellowish-natural | "Sandwich" effect | Relatively unburned | Longitudinal |
| 2 | Brown, reddish-brown | Long bones: Higher temperature outside and lower inside | <50% carbonization | Transverse |
| 3 | Brown and black | Long bones: Lower temperature outside and higher inside | >50% carbonization | Thumbnail |
| 4 | Black (completely charred) | Cranium: Higher temperature outside and lower inside | <50% calcination | Step |
| 5 | Dark grey/Taupe grey | Cranium: Lower temperature outside and higher inside | >50% calcination | Delamination |
| 6 | Light grey | Cranium: Suture with a different coloration |  | Patina |
| 7 | Bluish grey and white/grey | Heat line |  |  |
| 8 | White/grey and orange/beige | Others |  |  |
| 9 | White |  |  |  |
| 10 | Mixed: Brown/dark grey and light grey/white |  |  |  |
